# Neuropsychiatric Adverse Events of Montelukast: An Analysis of Real-World Datasets and drug−gene Interaction Network

**DOI:** 10.3389/fphar.2021.764279

**Published:** 2021-12-20

**Authors:** Ryogo Umetsu, Mizuki Tanaka, Yoko Nakayama, Yamato Kato, Natsumi Ueda, Yuri Nishibata, Shiori Hasegawa, Kiyoka Matsumoto, Noriaki Takeyama, Kazuhiro Iguchi, Hiroyuki Tanaka, Eiichi Hinoi, Naoki Inagaki, Masatoshi Inden, Yoshinori Muto, Mitsuhiro Nakamura

**Affiliations:** ^1^ Laboratory of Drug Informatics, Gifu Pharmaceutical University, Gifu, Japan; ^2^ Laboratory of Pharmacology, Gifu Pharmaceutical University, Gifu, Japan; ^3^ Laboratory of Community Pharmacy, Gifu Pharmaceutical University, Gifu, Japan; ^4^ United Graduate School of Drug Discovery and Medical Information Sciences, Gifu University, Gifu, Japan; ^5^ Laboratory of Medical Therapeutics and Molecular Therapeutics, Gifu Pharmaceutical University, Gifu, Japan; ^6^ Department of Functional Bioscience, Gifu University School of Medicine, Gifu, Japan

**Keywords:** montelukast, neuropsychiatric adverse events, food and drug administration adverse event reporting system, drug-gene intraction, protein-protein interaction, enrichment analysis

## Abstract

Montelukast is a selective leukotriene receptor antagonist that is widely used to treat bronchial asthma and nasal allergy. To clarify the association between montelukast and neuropsychiatric adverse events (AEs), we evaluated case reports recorded between January 2004 and December 2018 in the Food and Drug Administration Adverse Event Reporting System (FAERS). Furthermore, we elucidated the potential toxicological mechanisms of montelukast-associated neuropsychiatric AEs through functional enrichment analysis of human genes interacting with montelukast. The reporting odds ratios of suicidal ideation and depression in the system organ class of psychiatric disorders were 21.5 (95% confidence interval (CI): 20.3–22.9) and 8.2 (95% CI: 7.8–8.7), respectively. We explored 1,144 human genes that directly or indirectly interact with montelukast. The molecular complex detection (MCODE) plug-in of Cytoscape detected 14 clusters. Functional analysis indicated that several genes were significantly enriched in the biological processes of “neuroactive ligand–receptor interaction.” “Mood disorders” and “major depressive disorder” were significant disease terms related to montelukast. Our retrospective analysis based on the FAERS demonstrated a significant association between montelukast and neuropsychiatric AEs. Functional enrichment analysis of montelukast-associated genes related to neuropsychiatric symptoms warrant further research on the underlying pharmacological mechanisms.

## Introduction

Montelukast is a selective leukotriene receptor antagonist used to treat bronchial asthma and nasal allergy. Although montelukast is generally well tolerated, several clinical trials and post-marketing studies have reported serious neuropsychiatric adverse events (AEs) (Philip et al., 2009a; Philip et al., 2009b; Kelsay, 2009; Calapai et al., 2014; Haarman et al., 2017). The potential association between montelukast and suicidal behavior has previously been demonstrated based on the results of a literature search of MEDLINE, EMBASE, International Pharmaceutical Abstracts, and the Food and Drug Administration (FDA) adverse event reporting system (FAERS) (Schumock et al., 2011). This information has led the FDA to issue multiple warnings concerning an increased risk of neuropsychiatric AEs after taking montelukast and other leukotriene antagonists, including aggressive behavior, anxiety, depression, abnormal dreams, excitement, hallucinations, insomnia, irritability, and potential suicidality (Marchand et al., 2013; Perona et al., 2016; Merck & Co, 2020). On March 4, 2020, the United States FDA issued a safety announcement regarding the necessity of boxed warnings about serious neuropsychiatric AEs for montelukast (Singulair) (Food and Drug Administration 2020).

The FAERS is a spontaneous reporting system (SRS) involving reports of AEs in a real-world setting that are voluntarily submitted by healthcare professionals, pharmaceutical companies, and patients. The FAERS database is publicly available, can be downloaded from the FDA website (http://www.fda.gov), and is used in the post-marketing safety assessments of approved drugs. The objective of this study was to evaluate the association between neuropsychiatric AEs using well-established pharmacovigilance indices such as reporting odds ratio (ROR).

The pharmacological mechanisms causing neuropsychiatric alterations are currently unclear (Khalid et al., 2017). Most drugs act via interactions with several proteins encoded by different genes. An analysis of drug–gene interactions improved our understanding of drug toxicity (Ludovini et al., 2016). In recent years, integrated analysis using FAERS data and drug–gene interaction analysis data has been proposed as a method to expand our knowledge of AEs (Wu et al., 2016; Lin et al., 2017; Tanaka et al., 2021). To better understand the toxicological mechanisms underlying montelukast-associated neuropsychiatric AEs, we extracted a data set of human genes interacting with montelukast from public databases and constructed a drug–gene interaction network. Functional enrichment analysis of these genes was performed to elucidate the potential toxicological mechanisms of montelukast-associated neuropsychiatric AEs.

## Methods

### Data Source

Data from April 2004 to December 2018 were extracted from the FAERS database on the FDA website. The informatic structure of the FAERS database is based on the international safety reporting guidelines issued by the International Council on Harmonization (ICH), known as ICH E2B guidelines (U. S. Department of Health and Human Services, 2014). We integrated our database from the FAERS dataset using FileMaker Pro Advanced software (FileMaker, Inc., Santa Clara, CA, United States), according to the ASCII Entity Relationship Diagram, which is publicly available from the FDA website (https://www.fda.gov).

Following the FDA’s recommendation, we excluded duplicate reports of the same patient from different reporting sources from the analysis and extracted reports. Drugs in FAERS are classified into four categories: primary suspect drug (PS), secondary suspect drug (SS), concomitant (C), and interacting (I), according to the anticipated degree of involvement for AEs. Only reports with the PS drug code were included in this analysis.

### Definition of Adverse Events

AEs were coded with terms found in the Medical Dictionary for Regulatory Activities (MedDRA, https://www.meddra.org), which is the dictionary for terminology used in the FAERS database. This study relied on the definitions provided by MedDRA version 21.0. To evaluate montelukast-associated AEs, we utilized the system organ classes (SOCs) of “psychiatric disorders,” “general disorders and administration site conditions,” “nervous system disorders,” “respiratory, thoracic and mediastinal disorders,” and “gastrointestinal disorders” (Table 1). The preferred terms (PTs) related to each SOC are summarized in Table 1.

**TABLE 1:** Number of reports and reporting odds ratio related to montelukast in the FAERS (January 2004−November 2018).

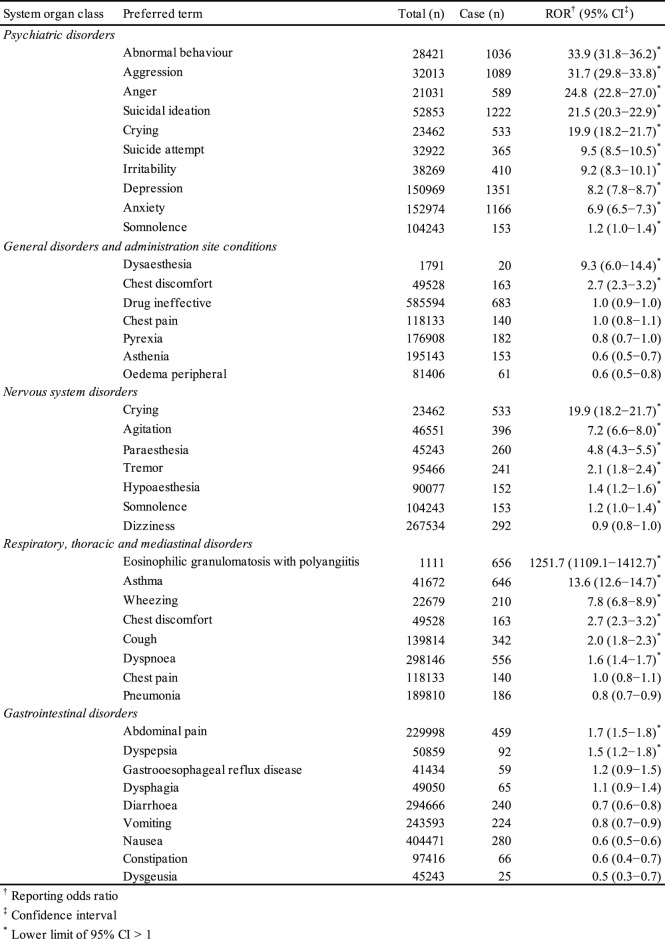

### Signal Detection

We used the ROR to analyze the association between montelukast and AEs. The ROR is the ratio of the odds of reporting an AE relative to all other AEs associated with the drug of interest compared with the reporting odds for all other drugs in the FAERS database (Poluzzi et al., 2012). ROR is calculated based on the two-by-two contingency table. RORs are expressed as point estimates with 95% confidence interval (CI). The signal was considered positive when the lower limit of 95% CI was >1 and the number of reports was ≥2 (Poluzzi et al., 2012).

### Drug−gene Interaction Network

The drug−gene interaction network was constructed on the basis of drug−gene and gene−gene interactions. Montelukast-associated genes were retrieved from DGIdb (drug–gene interaction database, https://www.dgidb.org), DSigDB (drug signatures database, http://dsigdb.tanlab.org), and STITCH (https://stitch.embl.de). The indirectly associated genes were retrieved from iRefIndex 15.0 (“9606. mitab,” https://irefindex.vib.be) (Razick et al., 2008). Molecular complex detection (MCODE) is an approach for detecting highly interconnected regions in protein−protein interaction networks (Bader and Hogue, 2003). The clusters likely to be involved in common biological function were investigated using the MCODE plug-in (version 1.5.1) of Cytoscape version 3.7 (http://cytoscape.org). This plug-in was utilized to choose hub modules of the gene−gene interaction network in Cytoscape with a degree cutoff = 2, node score cutoff = 0.2, k-core = 2, and Max. Depth from seed = 100 as the criteria. Next, we used “clusterProfiler (version 1.4.0),” an R package, to perform functional analysis and visualization of functional profiles for genes and gene clusters. We used the Kyoto Encyclopedia of Genes and Genomes (KEGG) enrichment analysis to explore the biological significance. KEGG enrichment analysis was performed using clusterProfiler with organism = “hsa,” pvalueCutoff = 0.05, pAdjustMethod = “BH,” and qvalueCutoff = 0.1. The thresholds in the KEGG enrichment analysis are pvalueCutoff = 0.05 and qvalueCutoff = 0.1. The default thresholds in the KEGG enrichment analysis are pvalueCutoff = 0.05 and qvalueCutoff = 0.2. Lin et al. applied pvalueCutoff = 0.05 and qvalueCutoff (not listed) (Lin et al., 2017). We could not find a gold standard for the thresholds. Finally, disease enrichment analysis based on DisGeNET was performed using the function enrichDGN in the R package named DOSE: Disease Ontology Semantic and Enrichment analysis (version 3.2).

## Results

The FAERS database contains 11,527,470 reports from January 2004 to December 2018. After excluding duplicates according to the FDA recommendations, 9,702,166 were analyzed. The RORs of suicidal ideation, suicide attempts, and depression in the SOC of psychiatric disorders were 21.5 (95% CI: 20.3–22.9), 9.5 (95% CI: 8.5–10.5), and 8.2 (95% CI: 7.8–8.7), respectively.

**FIGURE 1:**
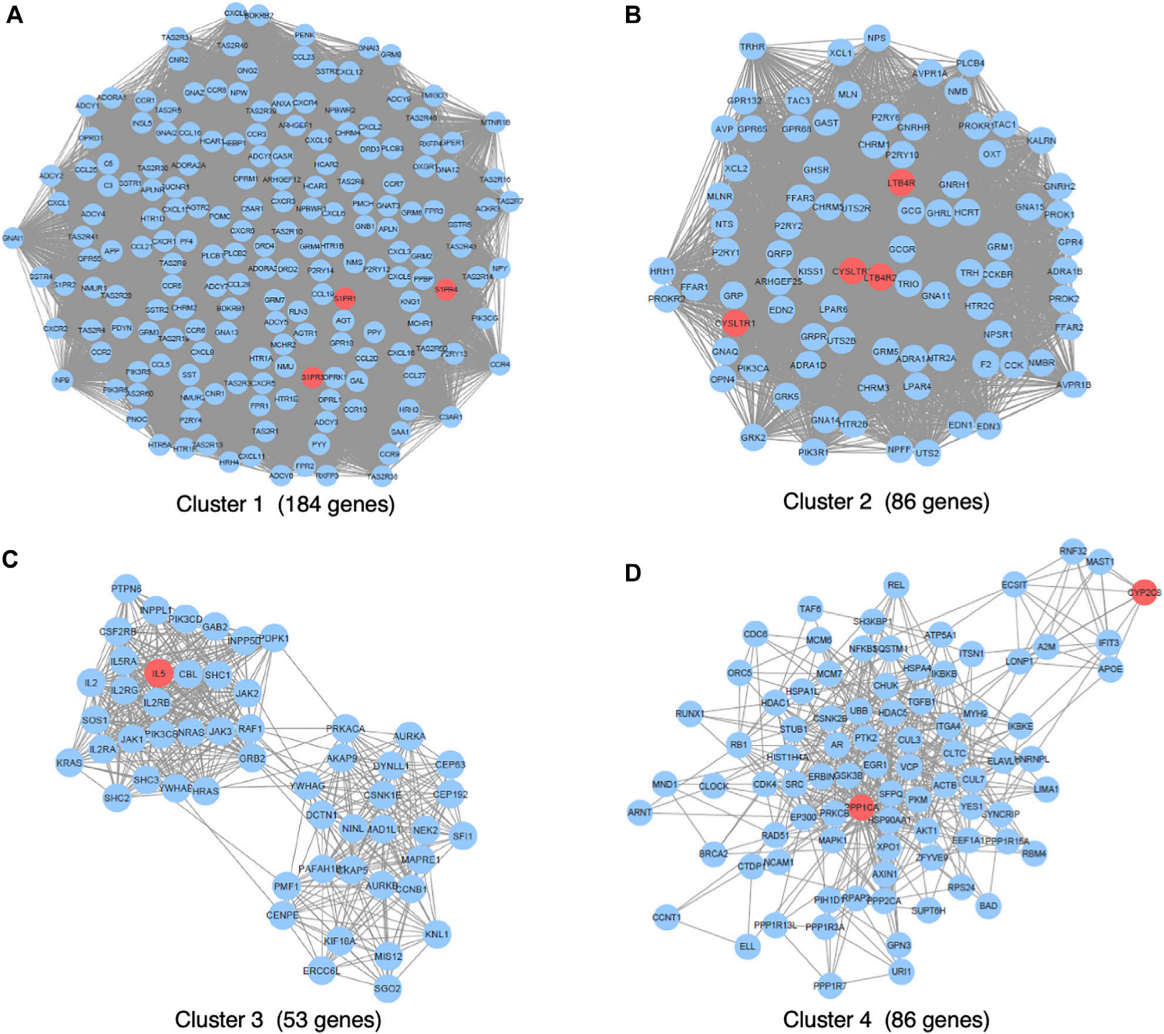
Top four gene interaction networks based on the MCODE plug-in of Cytoscape.

We primarily searched DGIdb (drug–gene interaction database, https://www.dgidb.org), DSigDB (drug signatures database, https://dsigdb.tanlab.org), and STITCH (https://stitch.embl.de) and retrieved 26 genes (ABCC1, AHR, ALOX5, ATAD5, ATG4B, CCL11, CYP2C8, CYSLTR1, CYSLTR2, IL13, IL4, IL5, KDM4A, LTA4H, LTB4R, LTB4R2, LTC4S, PLA2G1B, POLH, POLI, POLK, PPP1CA, S1PR1, S1PR3, S1PR4, SLCO2B) that interact with montelukast directly. All the genes of “9606. mitab” from iRefIndex 15.0 (https://irefindex.vib.be) were integrated into a network with 20,877 nodes and 429,350 edges. The genes that directly or indirectly interact with the above 26 genes were integrated into a network with 1,144 nodes and 35,384 edges. MCODE plug-in found 14 clusters (Table 2). To translate the network into biological insights, we further performed functional enrichment analysis using the KEGG pathways. The top four clusters stratified by biological process are shown in Figure 1 (Supplemental Figures 1A–D).

**TABLE 2:** Clusters of networks analyzed by MCODE.

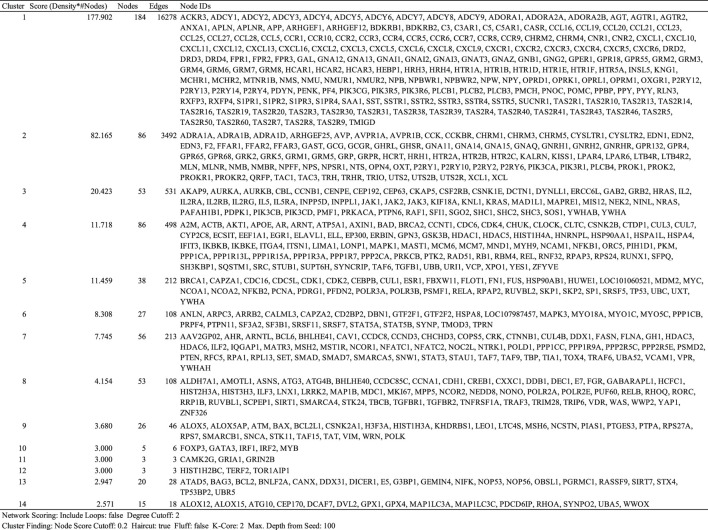

**FIGURE 2:**
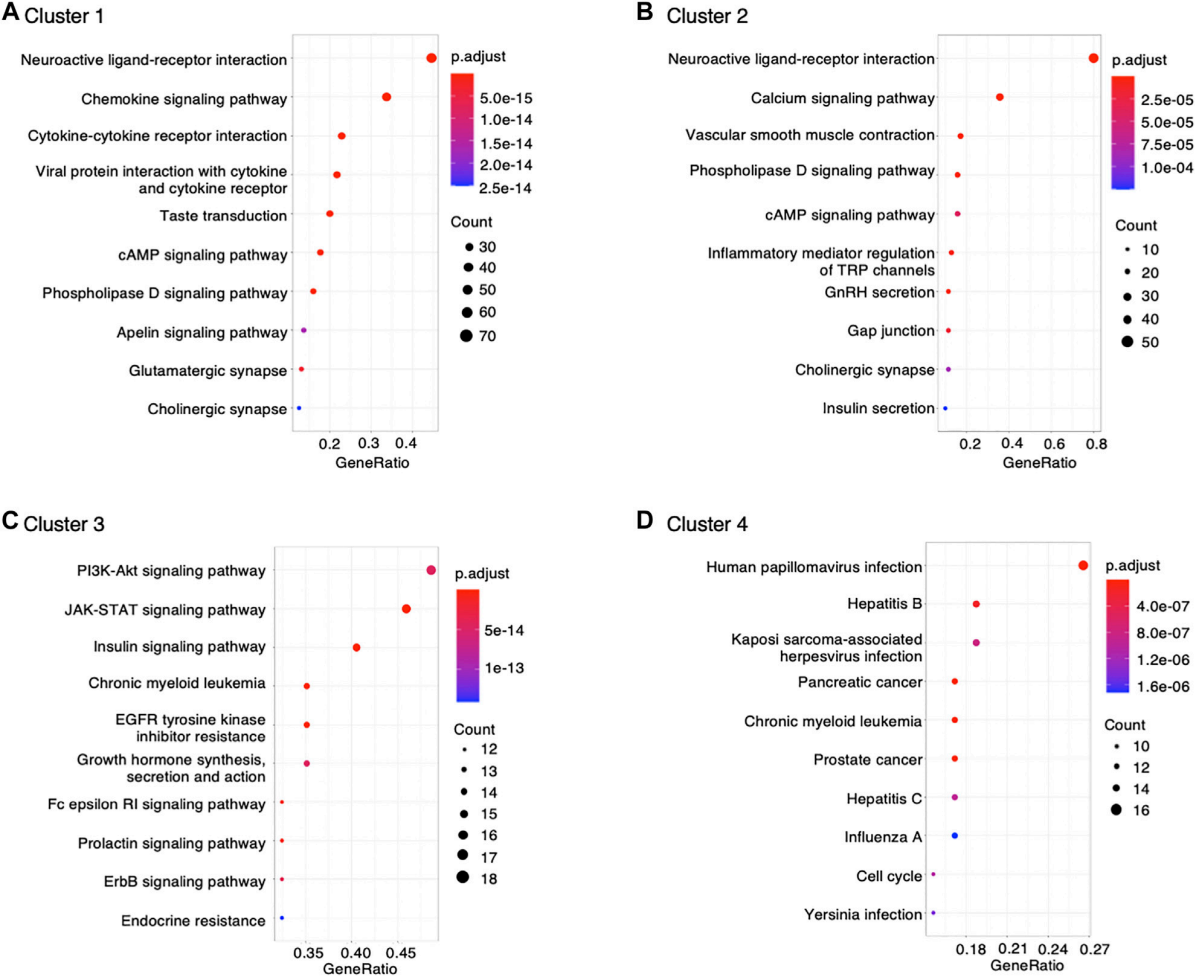
Dot plot of KEGG functional enrichment analysis.

The genes interacting with montelukast were enriched in a number of gene sets involved in “neuroactive ligand−receptor interaction” and “chemokine signaling pathway” in cluster 1, and “neuroactive ligand−receptor interaction” and “calcium signaling pathway” in cluster 2 (Figure 2).

**FIGURE 3:**
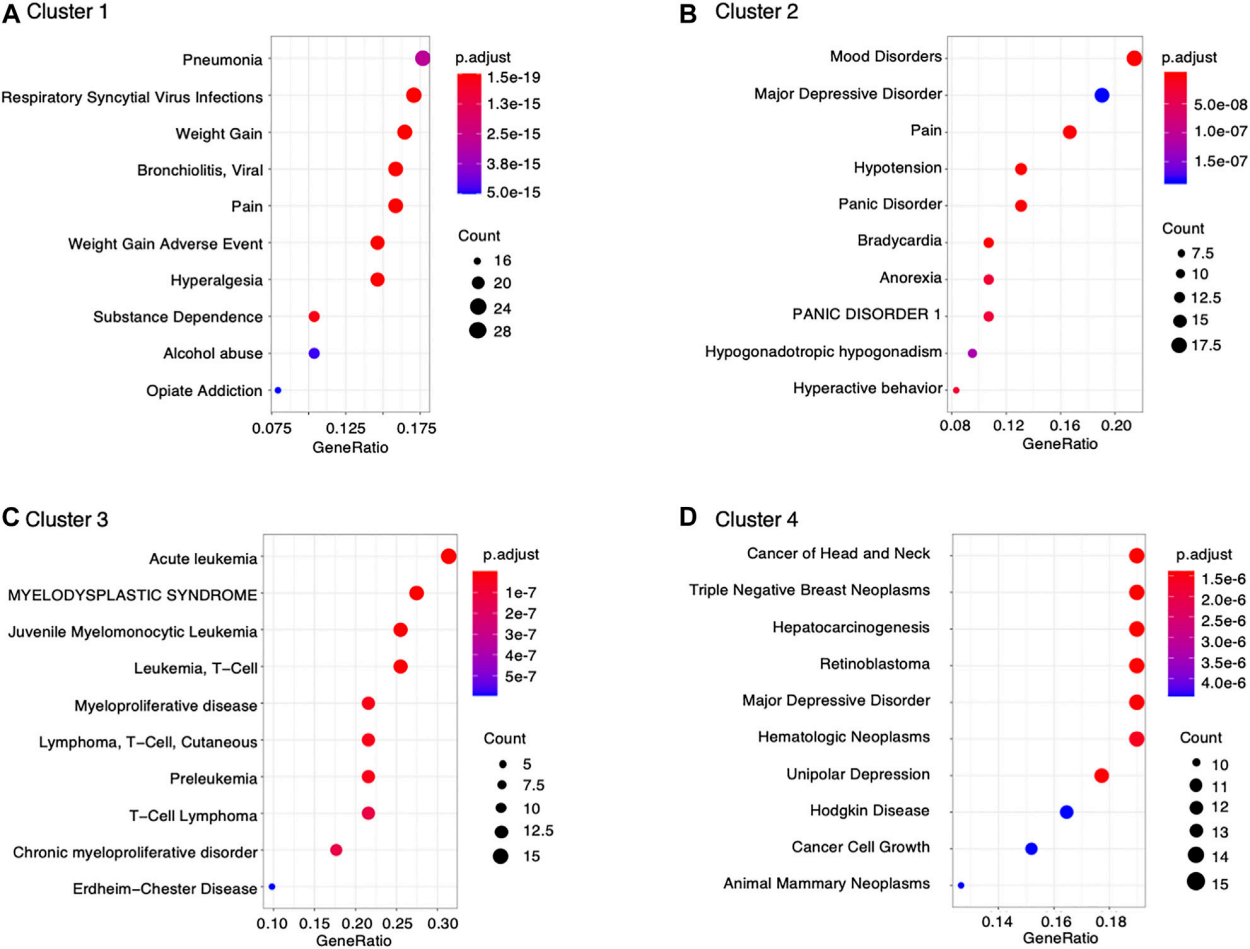
Dot plot of DisGeNET disease enrichment analysis.

Furthermore, data retrieved from DisGeNET was used to characterize diseases associated with montelukast. We found significant enrichment in genes involved in the following diseases related to montelukast (Figure 3): “pneumonia” (adjusted *p*-value = 2.85 × 10^–15^) and “respiratory syncytial virus infections” (adjusted *p*-value = 1.36 × 10^–17^) in cluster 1 and “mood disorders” (adjusted *p*-value = 1.21 × 10^–12^) and “major depressive disorder” (adjusted *p*-value = 1.83 × 10^–7^) in cluster 2.

## Discussion

We elucidated the AE profile of montelukast using the SOC of psychiatric disorders associated with the drug in the FAERS database. The lower limits of the 95% CIs of RORs related to the SOC of “psychiatric disorders” were more than 1, and the signal was significantly detected. Neuropsychiatric AEs have been found to account for the most important costs associated with comorbidity in asthma and have a negative impact on the patients’ quality of life (Schumock et al., 2011; Chen et al., 2016; Khalid et al., 2017), although most symptoms improve upon stopping montelukast therapy.

To better understand the toxicological mechanisms of montelukast-associated neuropsychiatric AEs, we curated drug−gene interactions from public databases. A total of 1,144 human genes interacting with montelukast were investigated. Some of these genes that were highly enriched in DisGeNET were related to “mood disorders” and “major depressive disorder” (Figure 3). HCRT (hypocretin neuropeptide precursor), HTR2A (5-hydroxytryptamine receptor 2A), and KALRN (kalirin RhoGEF kinase) genes were enriched in the modules “mood disorders” and “major depressive disorder” in cluster 2 (Table 2). HCRT encodes hypocretin, a hypothalamic neuropeptide precursor protein that gives rise to two mature neuropeptides, orexin A and orexin B, via proteolytic processing. The hypothalamic-pituitary-adrenal (HPA) axis plays an important role in the network mediated by stress-related neurotransmitters and have been proposed to affect depression (Bao et al., 2012) and suicide (Turecki et al., 2012). Hypocretins produced in the hypothalamus (Hunt et al., 2015) have functional interactions with the HPA axis and regulate sleep, feeding, energy balance, sexual behavior, and stress response, which are affected in depression (Nollet and Leman, 2013). HTR2A encodes 5-HT2A receptors, which are associated with major depressive disorder, schizophrenia, and suicidality (Niculescu et al., 2017). KALRN is a protein-coding gene that has been associated with stroke (Krug et al., 2010), coronary heart disease (Wang et al., 2007; Beręsewicz et al., 2008; Krug et al., 2010), schizophrenia (Hill et al., 2006; Hayashi-Takagi et al., 2010; Bradshaw and Porteous, 2012), and adult attention-deficit/hyperactivity disorder (Lesch et al., 2008). These findings suggest that montelukast could increase the risk of “psychiatric disorders.”

The common AEs caused by montelukast are upper airway infections, anaphylaxis, nausea, vomiting, diarrhea, elevated levels of liver enzymes, agitation, anxiety, depression, sleep disturbance, and eosinophilic granulomatosis with polyangiitis (EGPA), also known as Churg–Strauss syndrome (Calapai et al., 2014; Merck & Co, 2020). The presence of the ROR signal of EGPA in our study indicates the association of EGPA with the use of montelukast. However, the hypothesis that EGPA is not attributed to montelukast but to the reduction in the dose of glucocorticoid used in combination with montelukast has recently been accepted (Bibby et al., 2010). We considered that the value of the ROR related to EGPA was only apparently high.

Many studies supporting an association between leukotriene-modifying agents including montelukast and suicidality are primarily based on reviews of individual safety reports in AE databases which are subject to reporting bias and confounding factors. On the contrary, case-control and cohort studies, and clinical trials do not support an association between the two. Ecological studies have demonstrated a lack of positive association between leukotriene-modifying agents and suicidality at the population level (Khalid et al., 2017). Although our study is based on the FAERS database, it also has some limitations that are worth mentioning. As the FAERS is an SRS, it has several limitations including biases (under-reporting, over-reporting, missing data, and comorbidities), a lack of detailed information about the patients, and the exclusion of healthy individuals as a reference group. Therefore, ROR cannot be used for assessing true risks and ranking AEs. The risk of suicidal behavior increases among patients with respiratory diseases such as asthma (Schumock et al., 2011; Khalid et al., 2017). It has been elucidated how co-morbidities render FAERS data difficult to interpret (compared with controlled study data). Another limitation is that the SOCs in the FAERS data analysis and “mood disorders” and “major depressive disorder” in drug–gene analyses might not exactly represent the same clinical outcomes. Therefore, our results from the FAERS database must be interpreted considering these limitations. Further epidemiological studies using a large number of patients and well-controlled trials are required to confirm the safety risks of montelukast. When prescribing montelukast, clinicians should carefully monitor patients who may be at elevated risk for suicidal ideation or depression, according to the boxed warnings.

Some limitations of our functional enrichment analysis should also be noted. Our results do not offer any hard evidence regarding the potential mechanisms of montelukast-associated neuropsychiatric AEs. For now, the drug–gene interactions investigated have not been validated in any experimental model or *in vitro* and *in vivo* experiments because of our currently limited knowledge about disease-associated proteins and their interactions. Therefore, the association between montelukast and genes should be confirmed experimentally. Furthermore, we identified a list of 26 proteins interacting with montelukast. However, not all genes from databases like DGIdb are direct pharmacological targets; many may be indirectly affected by drugs. Our analysis uses a protein–protein interaction network to map these 26 affected proteins to larger networks and demonstrate that the networks are enriched in genes pertaining to mood disorders. We seized on the genes HCRT, KALRN, and HTR2A to substantiate the connection to mood disorders. As these genes are not direct targets of montelukast, this approach must be validated by showing that a given “hotspot” in such a protein–protein interaction network distinguishes drugs that cause “mood disorders” from drugs that do not. The modular assembly of drug safety subnetworks (MADSS) algorithm may be suitable for solving this problem (Lorberbaum et al., 2015).

## Conclusion

Our retrospective analysis demonstrated a significant association between montelukast and neuropsychiatric AEs. The genes that were thought to be associated with neuropsychiatric symptoms due to their interaction with montelukast were found to be significantly enriched in functional categories of psychiatric disease, which necessitates future pharmacological research.

## Data Availability

All datasets generated for this study are included in the article/Supplementary Material.
