# Supplementary figures and images for: Neuropsychiatric Adverse Events of Montelukast: An Analysis of Real-World Datasets and drug−gene Interaction Network
Source: Front Pharmacol. 2021 Dec 20;12:764279. doi: 10.3389/fphar.2021.764279 (PMC8720925; doi:10.3389/fphar.2021.764279)

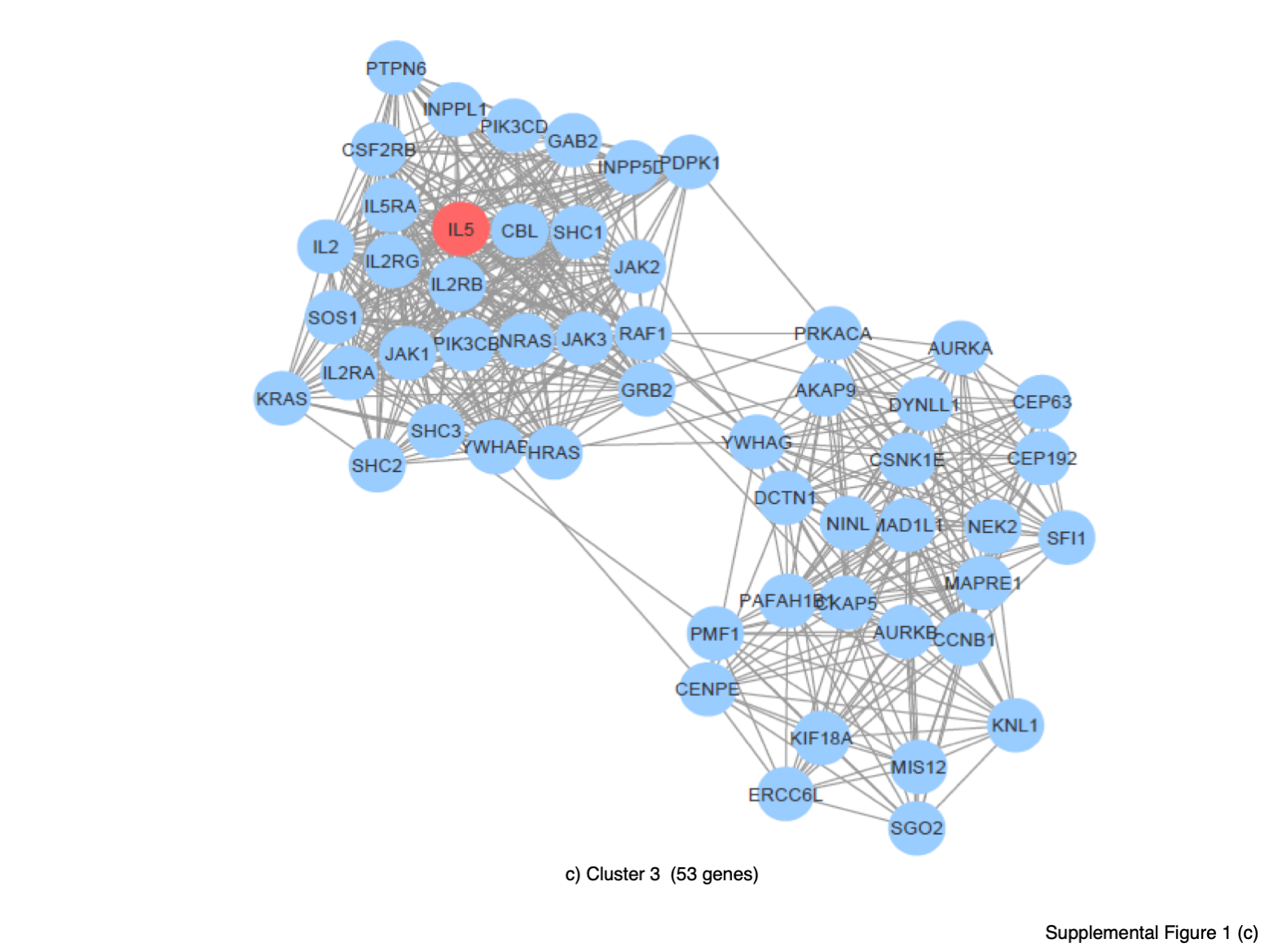

Supplement: Supplementary file 1 [file Image3.TIFF]

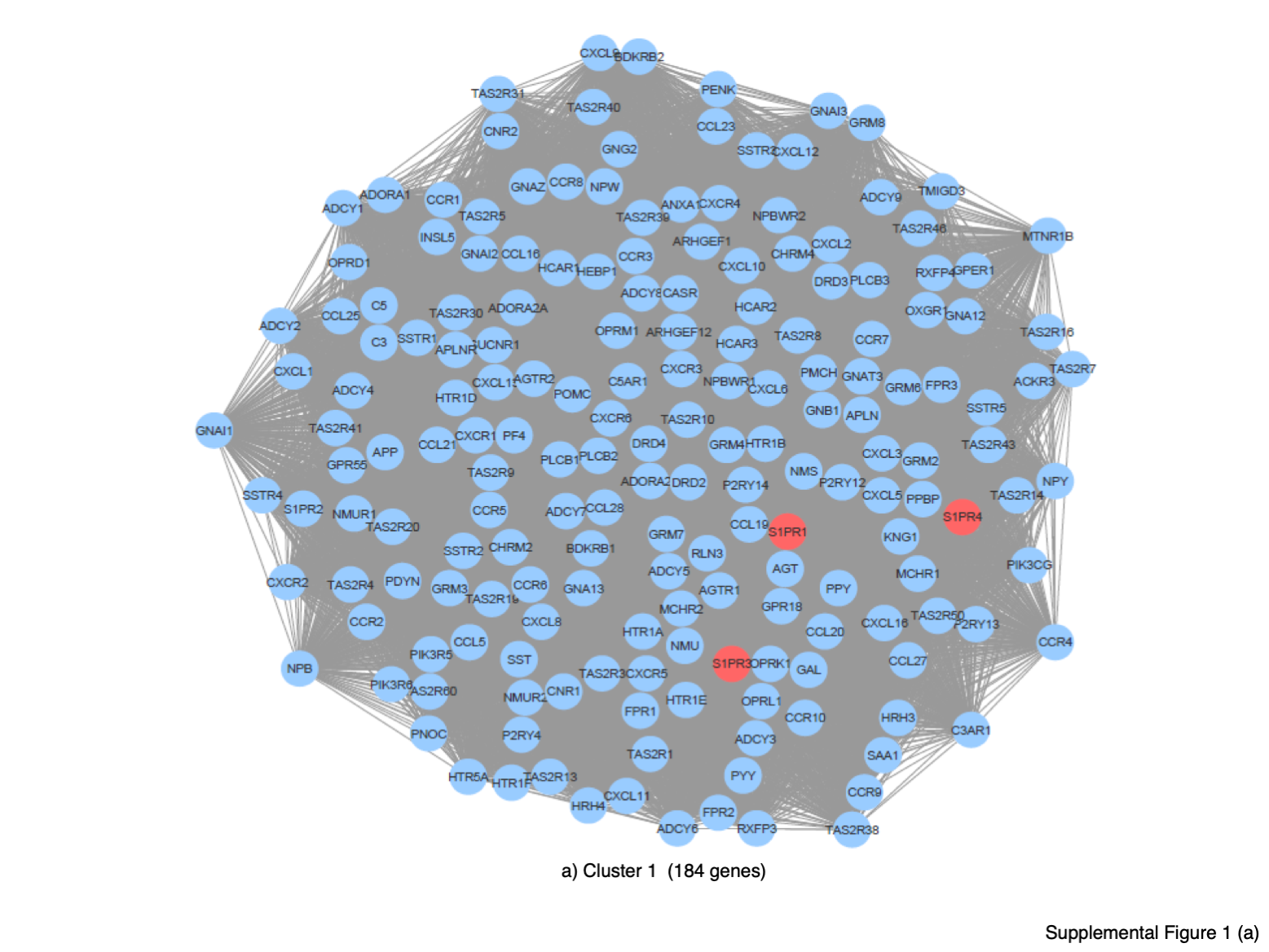

Supplement: Supplementary file 2 [file Image1.TIFF]

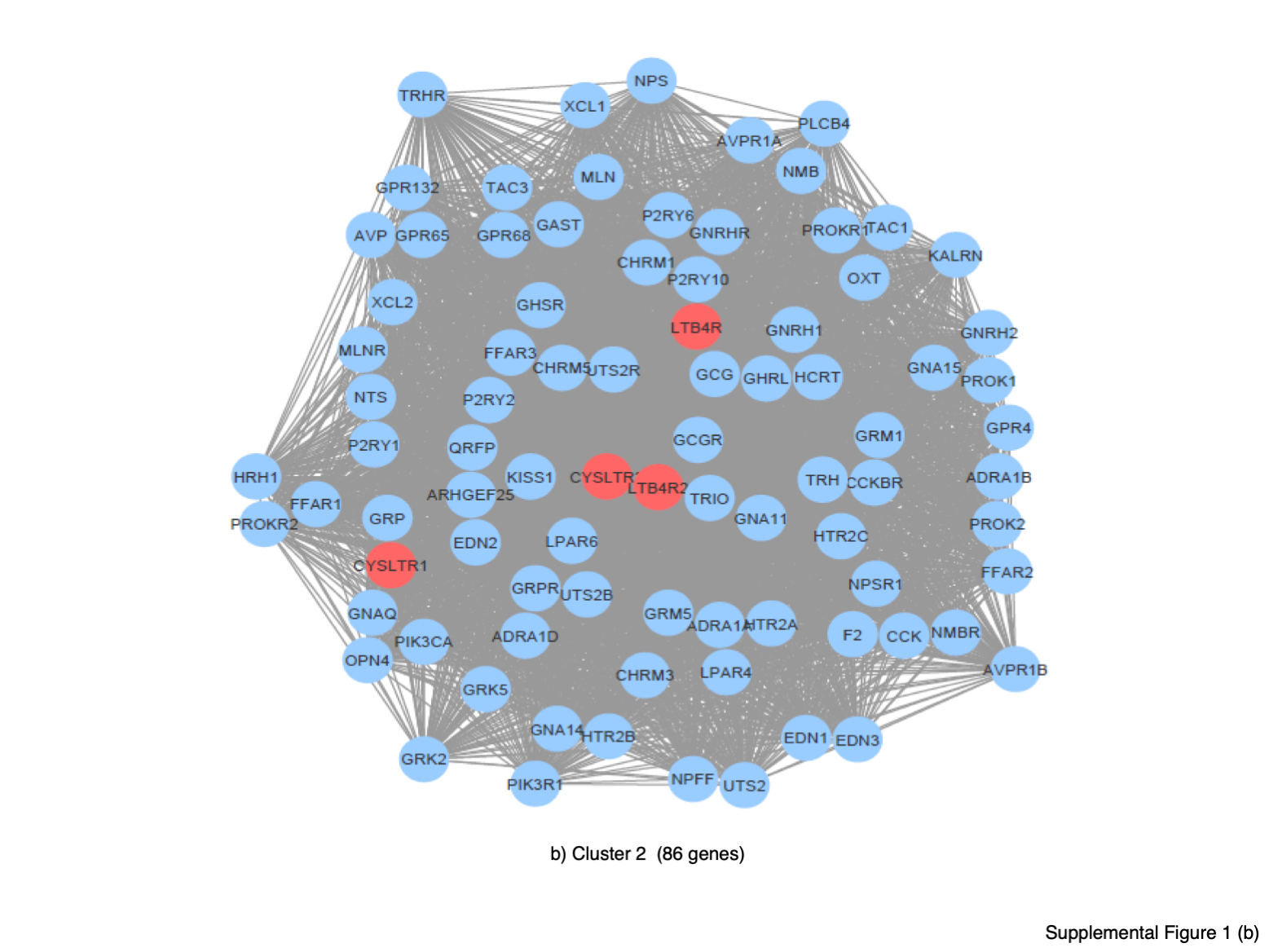

Supplement: Supplementary file 3 [file Image2.TIFF]

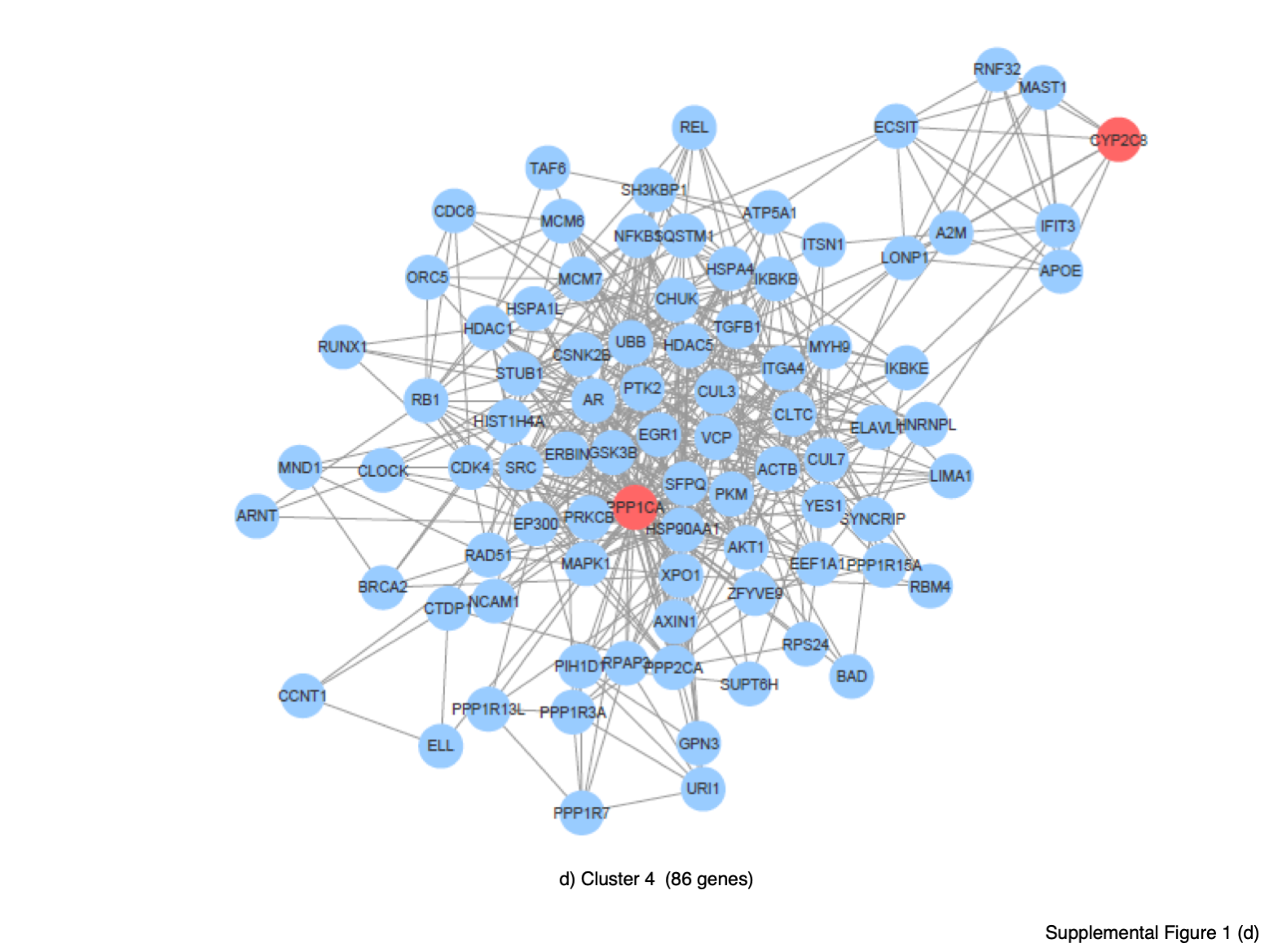

Supplement: Supplementary file 4 [file Image4.TIFF]
